# Hemoglobin A_1c_ improvements and better diabetes-specific quality of life among participants completing diabetes self-management programs: A nested cohort study

**DOI:** 10.1186/1477-7525-10-48

**Published:** 2012-05-14

**Authors:** Abhinav Khanna, Amber L Bush, J Michael Swint, Melissa Fleschler Peskin, Richard L Street, Aanand D Naik

**Affiliations:** 1 Baylor College of Medicine, 1 Baylor Plaza, Houston, TX, 77030, USA; 2 Houston Health Services Research and Development, Center of Excellence, Michael E. DeBakey VA Medical Center (152), 2002 Holcombe Blvd, Houston, TX, 77030, USA; 3 University of Texas School of Public Health, 1200 Hermann Pressler, Room E933, Houston, TX, 77030, USA; 4 University of Texas School of Public Health, 7000 Fannin, Suite 2648, Houston, TX, 77030, USA; 5 Department of Communication, Office: 205C Bolton, Texas A&M University, College Station, TX, 77843-4234, USA

**Keywords:** Diabetes-specific quality of life, Diabetes, Quality of life, Diabetes-39, Self-management, Hemoglobin A_1c_

## Abstract

**Background:**

Numerous primary care innovations emphasize patient-centered processes of care. Within the context of these innovations, greater understanding is needed of the relationship between improvements in clinical endpoints and patient-centered outcomes. To address this gap, we evaluated the association between glycosylated hemoglobin (HbA_1c_) and diabetes-specific quality of life among patients completing diabetes self-management programs.

**Methods:**

We conducted a retrospective cohort study nested within a randomized comparative effectiveness trial of diabetes self-management interventions in 75 diabetic patients. Multiple linear regression models were developed to examine the relationship between change in HbA_1c_ from baseline to one-year follow-up and Diabetes-39 (a diabetes-specific quality of life measure) at one year.

**Results:**

HbA_1c_ levels improved for the overall cohort from baseline to one-year follow-up (*t* (74) = 3.09, *p* = .0029). One-year follow up HbA_1c_ was correlated with worse overall quality of life (*r* = 0.33, *p* = 0.004). Improvements in HbA_1c_ from baseline to one-year follow-up were associated with greater D-39 diabetes control (*β* = 0.23, *p* = .04) and D-39 sexual functioning (*β* = 0.25, *p* = .03) quality of life subscales.

**Conclusions:**

Improvements in HbA_1c_ among participants completing a diabetes self-management program were associated with better diabetes-specific quality of life. Innovations in primary care that engage patients in self-management and improve clinical biomarkers, such as HbA_1c_, may also be associated with better quality of life, a key outcome from the patient perspective.

## Background

Diabetes Mellitus is among the most prevalent chronic illnesses in the United States, affecting nearly 24 million Americans [1]. In response to the Institute of Medicine’s calls for patient-centeredness [2], innovations in diabetes care have increasingly made patients’ perspectives central to the process and outcomes of care. These advances, which include the Chronic Care Model [3], the Patient-Centered Medical Home [4], and various patient-engagement interventions [5,6], all focus on patient-centeredness in the process of care. However, there is a need to move beyond the process of care and develop patient-centered outcomes to assess the impact of these innovations from the patient perspective.

As with many chronic diseases, diabetes patients are less concerned with clinical biomarkers [7] such as hemoglobin A_1c_, blood pressure, or lipid levels, and are more concerned with physical and social function, emotional and mental health, and the burden of illness and treatments on daily life [8]. Quality of life measures, which include many of these domains [9] are thus more meaningful and relevant outcomes from the patient perspective. The development of quality of life measures that are associated with future clinical outcomes would enhance shared decision making by framing treatment options in a context that is pertinent to patients [10].

In diabetes care, general health status measures such as the SF-36 and the EQ-5D are commonly used to assess patients’ quality of life [11-18]. Although these measures are useful in comparing patient health status across different illnesses, they often cannot capture distinctive aspects of specific diseases [19]. Quality of life measures that are disease-specific and associated with clinical outcomes have been developed in other chronic illnesses. For instance, a number of disease-specific quality of life measures in cardiovascular disease [10,20-22] and cancer [23-28] are predictive of subsequent morbidity and mortality. Diabetes places significant self-management responsibility on patients, and thus warrants the development and validation of clinically relevant and patient-centered quality of life measures. Recent structured reviews [9,29,30] have identified several disease-specific quality of life measures for diabetes. Unfortunately, attempts to understand the association between these quality of life measures and common clinical biomarkers, such as HbA_1c,_ have been inadequate [9,29,30].

We conducted a retrospective cohort study nested within a randomized comparative effectiveness trial of diabetes self-management interventions to investigate the association of HbA_1c_ and diabetes-specific quality of life. We evaluated the relationship between diabetes-specific quality of life and HbA_1c_ both before and after participants completed diabetes self-management programs.

## Methods

### Study design

This study was a retrospective cohort study nested within a pilot randomized comparative effectiveness trial conducted among diabetic patients at the Michael E. Debakey Veterans Affairs Medical Center (MEDVAMC). The trial randomized eligible participants to the Empowering Patients in Chronic Care (EPIC) goal setting intervention or to a diabetes self-management and nutrition education intervention. The primary study was conducted among 50–90 year old type II diabetes mellitus patients with primary care providers (PCPs) within the VA healthcare system. This secondary analysis included all participants from the original study who had HbA_1c_ ≥ 7.0% at baseline, completed either of the two self-management programs, had HbA_1c_ measurements in the VA clinic database at one-year follow-up, and returned completed one-year follow-up questionnaires.

### Diabetes self-management programs

All participants in our retrospective cohort completed one of two diabetes self-management programs. Both programs were conducted in group settings, included diabetes self-management education, and focused on educating participants about key clinical indicators in diabetes and the importance of integrating patient self-management into daily life. Each program included a ten minute one-on-one session with either a clinician or a diabetes educator to go over participants’ individual HbA_1c_, blood pressure, and cholesterol levels. The aim of the 1-on-1 personal sessions in both programs was to help participants individualize the diabetes self-management information. The Empowering Patients in Chronic Care (EPIC) intervention included didactic and problem-based discussions on goal-setting and action planning as well as patient-physician communication. The traditional diabetes education intervention included information on diabetes medications, associated health problems, meal preparation, and portion size and control. The complete methodology of the randomized comparative effectiveness study has been published elsewhere [6].

### Data collection

All data used in this study were collected during the EPIC pilot randomized comparative effectiveness trial after approval from the Baylor College of Medicine Institutional Review Board and the MEDVAMC research and development committee. No additional data were collected for this retrospective cohort study.

Clinical information, including hemoglobin A_1c_ and body mass index, was collected and then extracted from participants’ medical record in the MEDVAMC clinic database. Participants also completed questionnaires with a variety of self-reported data at baseline and one-year. Diabetes-related burden of illness was assessed at baseline as a proxy for diabetes-specific quality of life using a measure adapted from the Diabetes Care Profile Section VII [19]. This 13-item measure asks participants about aspects of their daily lives that diabetes interferes with, the burden of diabetes on personal finances, and how difficult life with diabetes is. Responses are along a 5-point Likert scale, with higher scores indicating a greater diabetes-related burden of illness. Individual diabetes burden of illness scores are calculated as the mean of all items, and thus range from 1–5. A co-morbidity score was determined using a measure derived from the Deyo modification of the Charlson Comorbidity Index [31].

### Diabetes-39: A diabetes specific quality of life questionnaire

Diabetes-related quality of life was assessed at one-year using the Diabetes-39 [32]. This 39-item self-administered instrument measures patients’ self-assessed quality of life, and includes 5 domains: diabetes control, anxiety and worry, social burden, sexual functioning, and energy and mobility. Respondents were asked “how much was the quality of your life affected by” a wide range of aspects of diabetes illness and its treatments in the past month. Possible responses are along a 7-point scale, and range from “Not affected at all” (=1) to “Extremely affected” (=7). Domain scores were calculated by summing the responses and then applying a linear transformation to a 0–100 scale. An overall quality of life score was calculated using all 39 items in the questionnaire. Scores closer to 0 indicate a better quality of life. The instrument has undergone tests for internal consistency (Cronbach’s alpha = 0.81–0.93; item-total correlation = 0.50–0.84), construct validity using the SF-36 Health Status Questionnaire, and a factor analysis, which found that five factors accounted for 90% of variance [33].

### Statistical analysis

We used descriptive statistics to characterize the cohort at baseline. Normality of continuous variables was assessed with Shapiro-Wilks test. Continuous variables were described using means, standard deviations, medians, and interquartile ranges, whereas categorical variables were described using counts and percents. Those who returned the follow-up survey at one year were compared to those who did not return the survey on demographic and clinical characteristics using Fishers Exact Test and the Wilcoxon Mann–Whitney test. The Wilcoxon signed rank sum test was used to compare change in HbA_1c_ from baseline to one-year follow-up and the Spearman Brown correlation (*r*_*sb*_) was calculated to assess the relationship between one-year HbA_1c_ and overall quality of life.

Multiple linear regression models were created to assess the relationships between change in HbA_1c_ from baseline to one-year and quality of life at one-year. Change in HbA_1c_ was calculated by subtracting baseline scores from 1 year scores. Therefore, higher scores indicated less improvement in HbA_1c_. Six regression models were conducted to separately predict the overall quality of life score and each of the five quality of life subscales. Treatment group (where diabetes education = 0 and EPIC intervention = 1) and baseline burden of illness were included as covariates in all six models. Because baseline Diabetes-39 quality of life scores were not available, baseline burden of illness served as a proxy for baseline quality of life. All analyses were performed using SAS software, version 9.2 (SAS Institute Inc, Cary, NC).

## Results

### Sample characteristics

We identified a cohort of participants completing one of two diabetes self-management programs as part of a randomized comparative effectiveness trial. The current study draws from the 94 participants who were consented and enrolled as part of the original comparative effectiveness trial. We excluded 14 participants from our cohort who did not return one-year follow-up questionnaires. Four additional participants were excluded for having baseline HbA_1c_ below 7.0%, and one participant was excluded due to an incomplete D-39 questionnaire. A total of 75 participants were included in the analytical cohort for this study.

Table 1 describes the baseline characteristics of the study cohort. Those with baseline HbA1c of at least 7.0% who did not return the follow-up questionnaire (n = 11) were not significantly different from study includes (n = 75) on any of the demographic or clinical characteristics reported in Table 1 (all *p*s > .05, data for non-respondents not reported). The cohort was predominantly older men of diverse education and racial/ethnic backgrounds, with multiple morbidities and elevated BMI and HbA_1c_ levels at baseline.

**Table 1:** Descriptive Statistics for N = 75 Patients

	***Mean (SD)***	***Median (IQR)***
Age in years	64.03 (7.56)	63.00 (9.00)
Baseline Burden of illness^b^	2.61 (0.81)	2.77 (1.30)
Baseline Body mass index *(n = 66)*	33.38 (5.86)	32.16 (5.59)
Deyo comorbidity index (n = 71)	3.66 (2.55)	3.00 (3.00)
Hemoglobin A1c %		
Baseline	8.82 (1.20)	8.60 (1.40)
1-year	8.28 (1.40)	7.90 (1.40)
1-year Diabetes QOL		
Overall	41.36 (23.32)	40.33 (35.05)
Diabetes control	42.24 (26.90)	43.06 (48.62)
Anxiety/worry	37.94 (30.25)	37.50 (58.34)
Social burden (n = 74)	23.96 (26.32)	13.33 (40.00)
Sexual functioning (n = 74)	60.65 (34.34)	66.67 (50.00)
Energy and mobility	43.10 (25.83)	43.33 (41.11)
	*Frequency (Percent)*	
Male	73 (97.33)	
Race/Ethnicity *(n = 74)*		
White	38 (51.35)	
Black	21 (28.38)	
Hispanic	12 (16.22)	
Other	3 (4.05)	
Education Level^a^		
≤ High School	21 (28.00)	
Some College/Trade School	54 (72.00)	
VA co-pay		
Required	25 (33.33)	
Waived	50 (66.67)	

^a^ Highest completed education level.

^b^ Adapted from a subscale of the Diabetes Care Profile (Fitzgerald 1996).

SD = standard deviation; IQR = inter-quartile range.

### Outcome measures

Significant improvements in HbA_1c_ levels were observed for the cohort from baseline to one-year follow-up, *S* = −574, *p* = .001. At follow-up, mean scores for overall quality of life and diabetes control, anxiety and worry, and energy and mobility subscale scores were similar to each other. Social burden subscale scores were better than overall quality of life scores, while sexual functioning subscale scores were worse than overall quality of life scores.

Higher one-year HbA_1c_ scores were associated with worse overall quality of life (*r*_*sb*_ = 0.37, *p* = 0.001). We conducted a series of six multiple linear regression models to assess the relationship between change in HbA_1c_ from baseline to one-year and Diabetes-39 quality of life (overall and for each subscale) at one-year. Results are presented in Table 2, with all models adjusting for burden of illness at baseline and treatment group. Irrespective of intervention group assignment and baseline burden of illness, improved HbA_1c_ levels from baseline to one-year follow-up were significantly associated with greater quality of life on the diabetes control (*β* = 0.23, *p* = .04) and sexual function subscales (*β* = 0.25, *p* = .03). Change in HbA_1c_ from baseline to one-year was not associated with greater overall quality of life or the anxiety/worry, social burden, or energy and mobility subscales. The *R*^2^ values for the diabetes control, sexual function, and energy and mobility subscale models were significant, indicating that these models explain a significant amount of the variability in their respective diabetes-specific quality of life subscales.

**Table 2:** Multiple Regression Models Predicting Overall QOL and QOL Subscales at One Year (N = 75)

	**Overall QOL**^**a**^		**Diabetes Control**		**Anxiety/ Worry**		**Social Burden**		**Sexual Function**		**Energy and Mobility**	
	***β***	***p***	***β***	***p***	***β***	***p***	***β***	***p***	***β***	***p***	***β***	***p***
Change in Hemoglobin A1c (baseline – 1 year) ^b^	.17	.13	.23	.04	.10	.42	.10	.40	.25	.03	.05	.64
Covariates												
Treatment Condition ^c^ (0 = DM education; 1 = EPIC)	-.05	.68	-.04	.74	-.04	.71	-.05	.70	-.03	.81	-.07	.54
Baseline Burden Inventory	.36	.002	.37	.001	.27	.019	.24	.04	.24	.04	.35	.003
*R*^*2*^	.18**		.21**		.10		.08		.14*		.14*	

^a^ Lower scores indicate better quality of life.

^b^ Lower scores indicate more improvement.

^c^ Treatment Condition = randomization to either Diabetes Mellitus (DM) Education Group Intervention or Empowering Patients in Chronic care (EPIC) Intervention.

* p < .05, ** p < .01.

## Discussion

We constructed a retrospective cohort of participants drawn from a randomized comparative effectiveness study to evaluate the relationship between change in HbA_1c_ and Diabetes-39 quality of life. HbA_1c_ at one-year follow-up was significantly associated with overall quality of life on the Diabetes-39. Our multiple linear regression models suggest that improvements in HbA_1c_ among patients completing diabetes self-management interventions are significantly associated with increased quality of life on the diabetes control and sexual functioning subscales of the Diabetes-39. No association was established between changes in HbA_1c_ and the anxiety and worry, social burden, and energy and mobility subscales. Baseline burden of illness, a proxy for baseline quality of life, predicted overall quality of life as well as all subscales of the Diabetes-39, as expected.

This study firmly establishes the relationship between improved HbA_1c_, a critical clinical biomarker in diabetes, and the Diabetes-39, a patient-centered diabetes-specific quality of life measure among patients completing a self-management education program. Several previous studies have attempted to explore the relationship between clinical indicators, such as HbA_1c_, and a variety of diabetes-specific quality of life measures [31,34-40]. Unfortunately, these associations have been weak [41] or nonexistent [42], present for only very few of a scale’s domains [36], or are specific to type 1 diabetes only [34,35]. Further, prior studies report on measures that have poor evidence for validity and reliability [32,35,36,41], focus on singular aspects of quality of life (e.g., distress [37,38,41]), ignore key components of quality of life such as physical and social functioning [9], or include several items that are not diabetes-specific [9]. Additionally, several reviews of diabetes-specific quality of life measures [9,29,30] have recognized the lack of empirical evidence on the responsiveness of these scales to changes in health status.

This analysis of HbA_1c_ and diabetes-specific quality of life addresses many of the limitations of prior studies. The Diabetes-39 diabetes-specific quality of life measure has been recommended for use in research and clinical settings by all of the aforementioned reviews of diabetes-specific quality of life measures [9,29,30]. The instrument has good evidence for validity and reliability, includes several domains that cover many aspects of quality of life, and is applicable to a wide population of patients [9,29,30,33]. The Diabetes-39 is one of few diabetes-specific quality of life measures that have been shown to be responsive to changes in health status [39]. Further, this instrument does not impose a definition of quality of life upon respondents, but instead allows patients to frame responses in the context of their own personal conceptualization of quality of life. Also, patients were directly involved in the selection of items for the questionnaire [33]. These attributes make the instrument highly patient-centered, one of the most critical components to any patient-assessed quality of life measure. Thus, our study focuses on a diabetes-specific quality of life measure that is a prime candidate for analysis.

Our statistical methods also address several prior studies’ shortcomings. While most previous attempts to examine the relationship between HbA_1c_ and quality of life used simple linear correlations [34,41,42], our analyses included predictive linear regression models. This allows for a more robust analysis and provides a quantification of the impact of HbA_1c_ on quality of life. To our knowledge, two prior studies have employed linear regression models to assess this relationship [37,38]. However, one study [38] grouped continuous HbA_1c_ data into two groups. This reduces a model’s ability to quantify the effect of changes in HbA_1c_ on quality of life, and diminishes the overall robustness of the model. A second study [37] modeled HbA_1c_ as the primary dependent variable. This is not in line with the Institute of Medicine’s vision [2] in which patient-centered measures, such as quality of life, are the ultimate outcomes of care. Our analysis included a regression of continuous HbA_1c_ data with quality of life as the primary outcome.

Few prior studies have examined the relationship between clinical indicators and diabetes-specific quality of life measures among participants who all completed diabetes self-management programs. These programs were deeply embedded in primary care. One program was led by a primary care physician, while the other was led by nurse educators and registered dieticians. The latter model represents the type of delivery system redesign that is characteristic to many primary care innovations [3,4]. Our examination of the relationship between clinical indicators and quality of life outcomes in the context of patient-centered diabetes self-management programs demonstrates that HbA_1c_ improvements among participants in these programs are associated with better quality of life. Previous studies have included diabetes-specific quality of life among outcome measures [40,43]. These studies approach both quality of life and HbA_1c_ as distinct outcomes, and do not explore the association between the two variables. Unlike prior studies, our study examines the relationship between changes in HbA_1c_ and diabetes-specific quality of life. In the post-ACCORD era, there has been reduced emphasis on intensive HbA_1c_ control [44]. However, the current study suggests that improved HbA_1c_ resulting from diabetes self-management interventions is associated with better diabetes-specific quality of life. Thus, HbA_1c_ control is relevant to patient-centered outcomes and should remain a valuable goal in diabetes care.

There were limitations to our study. A sample size of 75 limited the range of analytic strategies that could be employed. The sample size may also have affected the power of our analyses, which may account for the weak association between changes in HbA_1c_ and some of the Diabetes-39 subscales. The generalizability of our study may also be limited. Our sample is reflective of the United States Veterans Administration patient population, consisting largely of older patients who are predominantly male, of older age, and have significant co-morbidities. Further, all of the participants in our cohort participated in at least one diabetes self-management program. Thus, we were unable to assess the impact of participation in these programs on quality of life as compared with patients who did not participate in any self-management programs. Additionally, the lack of Diabetes-39 data at baseline precluded an examination of the responsiveness of this diabetes-specific quality of life measure over time. However, our analysis does include HbA_1c_ data from multiple time points and includes a measure of burden of illness at baseline. Many previous studies used cross-sectional data from one time point [34,37,41,42]. Our analyses included HbA_1c_ data from both before and after participation in diabetes self-management programs.

Future studies should be certain to collect quality of life data both before and after diabetes self-management programs so that the responsiveness of quality of life measures can be assessed. Subsequent studies should also include larger, more diverse samples to ensure adequate power and generalizability. The inclusion of a control group that does not receive any programs beyond routine care may also allow for future examinations of the impact of diabetes-self management programs on quality of life.

## Conclusion

Improved HbA1c levels among participants in diabetes self-management programs are associated with higher diabetes-specific quality of life scores. These findings suggest that innovations in primary care focused on patient engagement may not only improve traditional clinical outcomes, but are associated with better patient-centered quality of life outcomes.

## Competing interests

The authors declare that they have no competing interests.

## Authors’ contributions

AK and AN conceived of the study and participated in its design. AK drafted the manuscript. AB conducted statistical analysis and helped draft the manuscript. AN participated in study conception and design, helped draft the manuscript, and conducted the parent study from which data for this study was derived. JS, RS, and MP contributed to interpretation of data, revising manuscript critically, and final approval of the version submitted to the journal. All authors read and approved the final manuscript

## Authors’ information

AK is an MD/MPH candidate at Baylor College of Medicine and the University of Texas School of Public Health. AN is the an investigator in the Health Decision-Making and Communication Program, Houston VA Health Services Research and Development CoE, Michael E. DeBakey VA Medical Center. AN is also an Assistant Professor, Department of Medicine, Section of Health Services Research, Baylor College of Medicine and Adjunct Assistant Professor, Division of Health Promotion and Behavioral Sciences, School of Public Health, The University of Texas Health Sciences Center at Houston. AB is a Programmer in the Design and Analysis Program, Houston VA Health Services Research and Development CoE, Michael E. DeBakey VA Medical Center and an Instructor in the Department of Medicine, Section of Health Services Research, Baylor College of Medicine. JS is a Professor of Health Economics, at the University Texas School of Public Health and at the University of Texas Medical School, Center for Clinical Research Evidence-Based Medicine, and Adjunct Professor, Department of Economics, Rice University. RS is a Research Professor in Medicine at Texas A&M University and Director, Health Communication and Decision-Making Program in the Houston Center for Quality of Care and Utilization Studies, Baylor College of Medicine. MP is the Associate Director of Evaluation for the University of Texas Prevention Research Center and an Assistant Professor at The University of Texas School of Public Health.
